# A New Function of the DRD1 Gene: GnRH Secretion Regulation in Sheep Hypothalamic Neurons

**DOI:** 10.3390/genes16030273

**Published:** 2025-02-25

**Authors:** Manjun Zhai, Shaoqi Cao, Huihui Liang, Yifan Xie, Zongsheng Zhao

**Affiliations:** 1 College of Animal Science, Xinjiang Agricultural University, Urumqi 830052, China; 2 Xinjiang Uygur Autonomous Region Animal Husbandry General Station, Urumqi 830001, China; csqshzu@163.com (S.C.); xjzy0991@163.com (H.L.); 3 College of Animal Science and Technology, Shihezi University, Shihezi 832003, China; 18899593750@163.com (Y.X.); zhaozongsh@shzu.edu.cn (Z.Z.)

**Keywords:** Kazakh sheep, DRD1, hypothalamic, GnRH

## Abstract

Background: Dopamine (DA) is an important neurotransmitter that is widely present in the central nervous system. DA plays a crucial regulatory role in mammalian emotion, endocrine function, and reproduction through the activation of dopamine receptors. We compared the transcriptomes of hypothalamic tissues from Kazakh sheep during the nonbreeding season of anoestrus and during the nutrient-induced nonbreeding season of oestrus. Our research findings suggest that the dopamine receptor D1 (*DRD1*) gene may be a candidate gene for the regulation of sheep oestrus. However, the underlying mechanism through which *DRD1* regulates sheep oestrus is still poorly understood. Methods: In the present study, the expression of *DRD1* mRNA in the hypothalamus of oestrous Kazakh sheep was significantly greater than that in the anoestrous phase. Immunohistochemical staining revealed that *DRD1* was more widely expressed in hypothalamic tissue and was more highly expressed during oestrus than during anoestrus. Hypothalamic neuron experiments further indicated that *DRD1* affects the expression of GnRH through dopamine synapses and calcium signalling pathways. Results: moreover, the overexpression of the *DRD1* gene promoted the secretion of GnRH, while knocking down the *DRD1* gene reduced the secretion of GnRH. Conclusions: The present study revealed that the *DRD1* gene plays a crucial regulatory role in the secretion of the hormone GnRH in the hypothalamus of Kazakh sheep.

## 1. Introduction

Sheep farming is an extremely important component of China’s animal husbandry industry. Approximately one-third of the country’s regional sheep breeds are local sheep breeds, and Xinjiang has many excellent breeds. However, most excellent local sheep breeds, such as typical Kazakh sheep and Altay sheep, exhibit seasonal oestrus. In the lamb meat market, Xinjiang local sheep breeds are favoured by consumers due to their excellent meat quality and flavour, and these local sheep breeds, especially Kazakh sheep, have advantages such as tolerance to roughage, strong stress resistance, and strong adaptability. At present, the demand for sheep in the market is constantly increasing, and local sheep breeds in Xinjiang have low reproductive capacity and typical seasonal oestrous traits. One litter per year seriously restricts production efficiency. Therefore, seasonal oestrus in sheep is one of the key factors that currently restricts sheep farming production.

The *DRD1* gene is located on sheep chromosome 7 (OAR 7). *DRD1* (Gene ID: 100568292) has a 4120bp genomic sequence, in which a 95bp intron is excised to produce the mRNA that contains a 5′untranslated region plus the 1341 nt coding sequence (CDS) mRNA, and the open reading frame encodes 446 amino acids. The dopamine receptor D1 (DRD1) gene, a member of the G-coupled protein family, is expressed at high levels in hypothalamic tissue and can promote or inhibit the secretion of prolactin (PRL), which is closely related to the reproductive system [1]. Currently, research on the *DRD1* gene in domestic chickens and pigs has focused mainly on the correlation between genetic polymorphisms and reproduction [2,3,4]. In Thai chickens, there is a correlation between dopamine neurons and the regulation of the reproductive system [5], and hens can inhibit the secretion of PRL through dopamine receptor blockers or antagonists [6,7]. Furthermore, previous studies have shown that the fertility of homozygous *Drd1* gene knock-out mice is reduced in comparison to that of heterozygous animals, demonstrating the importance of Drd1 in reproduction [8]. These examples suggest that the *DRD1* gene may have additional roles that are important for reproductive ability.

To date, there have been no reports on the role of the *DRD1* gene in sheep oestrus. In our previous studies [9], transcriptome analysis suggested that *DRD1* might be involved in sheep oestrous regulation by regulating other reproduction-related genes. Therefore, the present study used real-time fluorescence quantification and immunohistochemistry to detect *DRD1* mRNA expression and protein expression in the hypothalamic tissues of Kazakh sheep in oestrus and in nutrient-induced oestrus. The effect of *DRD1* on GnRH secretion in hypothalamic neurons at the cellular level was revealed through gene overexpression and RNA interference (RNAi) methods, as was the regulatory effect of *DRD1* on the expression levels of the genes involved in sheep oestrus. This study aims to investigate the role of *DRD1* in GnRH secretion regulation and its expression differences in hypothalamic tissue during the oestrous and anoestrous phases in Kazakh sheep. This research reveals the regulatory role and molecular mechanism of *DRD1* in sheep oestrus.

## 2. Materials and Methods

### 2.1. Samples

#### Ethics Statement

Procedures were performed according to the *Guide for the Care and Use of Laboratory Animals*. The animal use protocol was reviewed and approved by the Institutional Animal Care and Use Committee of the First Affiliated Hospital of the Medical College of Shihezi University, Xinjiang, China (A2020-107-01). The 90-day-old female sheep foetus was obtained from a live animal slaughtering centre in Shihezi, Xinjiang. The hypothalamic, pituitary, and ovarian tissues were collected from 6 healthy adult Kazakh ewes that were 2–4 years old and 40–45 ± 1.5 kg in weight and were in oestrus and anoestrus, provided by the Experimental Station of Shihezi University, Xinjiang, China. All samples were collected by trained personnel and specialised veterinarians following the animal welfare protocols of the First Affiliated Hospital of the Medical College of Shihezi University.

### 2.2. Primer Design for RT-qPCR and mRNA Expression Analysis

The primers used were designed with Primer 5.0, and the sheep *DRD1* mRNA sequence was obtained from Ensembl, with β-actin used as an internal reference gene (Table 1). The primers used were synthesised by Sangon Biotech (Shanghai, China). Total RNA was extracted from hypothalamic, pituitary, and ovarian tissues using TRIzol [10], and a PrimeScriptTM RT reagent kit with gDNA Eraser (Tarak) was used for reverse transcription to obtain cDNA. The specific operating steps for the culture were performed according to the manufacturer’s instructions (20 °C). Real-time fluorescence quantitative technology was used to measure the mRNA expression levels of the *DRD1* gene in the hypothalamic, pituitary, and ovarian tissues, with 3 replicates in each group. The primer information is shown in Table 1. The real-time fluorescence quantitative PCR system was as follows: 10 μL SYBR GreenIMaster, 1 μL upstream and downstream primer, 1.5 μL cDNA, and 6.5 μL ddH2O, for a total volume of 20 μL. The reaction conditions were as follows: 95 °C, 600 s; 95 °C, 30 s; 54 °C, 30 s; 72 °C, 30 s; and 55 cycles.

### 2.3. Immunohistochemical Determination of the Distribution of the DRD1 Protein

The preparation and fixation of the tissue paraffin sections were conducted as follows: Hypothalamic, pituitary, and ovarian tissues were fixed in 4% paraformaldehyde solution for 48 h using the infiltration method. The sections were dehydrated by rinsing with running water and a gradient of alcohol to anhydrous ethanol. Xylene was then used to clear the sections, which were subsequently embedded in paraffin wax. Serial sections, 4 microns thick, were obtained from paraffin blocks. The sections were then incubated in a 62 °C oven for 2 h for later use.

Immunohistochemical staining was conducted as follows: The slices were reheated at 65 °C for 30 min and then dewaxed with xylene and a gradient of alcohol to water. Antigen retrieval was performed in citrate buffer (pressure cooker) for 8 min, followed by cooling to room temperature. The slices were subsequently placed in 3% H_2_O_2_ at room temperature in the dark for 10 min, rinsed 3 times with phosphate-buffered saline (PBS) for 5 min each time, and blocked with goat serum at room temperature for 30 min. The primary antibody (abcom, rabbit polyclonal to dopamine receptor D1, ab40653) was added (1:1000 was used as the optimal concentration after preliminary experiments were conducted to determine the concentration), followed by incubation overnight at 4 °C in a wet box and removal the next day for reheating at 37 °C for 30 min. The secondary antibody (zsbio, goat anti-rabbit IgG, Polymers, ZB-2306) was added, and the mixture was incubated at 37 °C for 30 min. The colour was visualised under a DAB chromogenic solution, and the sections were stained with a haematoxylin core for 1–2 min, rinsed with tap water, differentiated in alcohol for a few seconds, and rinsed again with tap water. Then, the sections were placed in the following, in order: 70% alcohol, 80% alcohol, 90% alcohol, 95% alcohol, alcohol, alcohol, xylene, and xylene. They were then air-dried and sealed with neutral gum. Note: after the addition of the primary antibody and secondary antibody and each staining procedure, the sections were soaked in PBS 3 times for 5 min each.

### 2.4. Design and Screening of siRNA DRD1 Interference Fragments

The target sequence of the *DRD1* gene (ENSOARG00020020572) was studied. The principles of RNA interference fragment design and the characteristics of the coding sequence of the sheep *DRD1* gene were used. The coding DNA sequence (CDS) region of the *DRD1* gene was obtained from the Ensembl database. The authors verified and screened the effects of siRNAs to design the fragments provided by siDirect. Preliminary screening was used to determine the siRNA target sequences, and the Basic Local Alignment Search Tool (BLAST) was used to analyse the homology of these sequences with other genes in the bodily tissues of the Kazakh sheep on the NCBI website to ensure that there was no interference with other genes (Table 2).

### 2.5. Constructing the Overexpression Vector pEGFP-C2-DRD1

The *DRD1* coding region for Kazakh sheep was synthesised by Shanghai Shenggong. The 1341 bp product (XhoI to BamHI) was ligated into the pEGFP-C2 plasmid to generate the pEGFP-C2-*DRD1* expression vector. The recombinant plasmid pEGFP-C2-*DRD1* was subjected to endotoxin-free large-scale extraction (Tiangen) and concentration measurements and was packaged at −20 °C for storage to await cell transfection.

### 2.6. Transfection of Plasmids into Hypothalamic Neurons

The sheep hypothalamic neurons in the present study were purified and cultured from approximately 90-day-old foetal sheep via collagenase digestion culture according to previous methods performed in our laboratory [11]. The animal experiments were performed in strict accordance with the guidelines for the care and use of laboratory animals of Shihezi University. Hypothalamic tissue was cut into pieces and digested in collagenase IV. Neurobasal medium-A (Gibco) supplemented with 2% B27 (Gibco) was added to the cell suspension, which was gently blown, mixed, and inoculated in polylysine-coated Petri dishes. After 48 h, the medium was replaced with neurobasal medium-A, B27, or 100X GlutaMAX at a ratio of 100:2:1 every 2 days. Neuronal identification was performed by PCR with TUBA4A/Noggin (Table 3), and immunofluorescence was performed with an anti-MAP2 antibody (primary antibody, abcam, rabbit monoclonal [EPR19691] to MAP2, 1:100; secondary antibody, CST, anti-rabbit IGg DyLight 680 Conjugate, 1:1000). pEGFP-*DRD1* was transfected into the following three groups of cells: NC, pEGFP-C2, and pEGFP-C2-*DRD1*. The siRNAs were transfected into the NC, siRNA-*DRD1*-1, siRNA-*DRD1*-2, and siRNA-*DRD1*-3 cells. After 24 h, the expression of green fluorescent protein (GFP) in the cells was observed under a fluorescence microscope (TE2000, Nikon, Tokyo, Japan) to determine the transfection efficiency. Three repeated experiments were performed by transfecting an equal number of cells with the same vector into different wells.

### 2.7. qRT-PCR Detection of the Impact of DRD1 on Genes Involved in Oestrus-Related Pathways

We used cDNA from the NC, pEGFP-C2-*DRD1*, and siRNA-*DRD1*-1 groups as templates. The *DRD1*, *GnAQ* (G protein subunit alpha q), *ITPR1* (inositol 1,4,5-trisphosphate receptor type 1), *PLCB1* (phospholipase C beta 1), and *PRKCB* (protein kinase C beta) mRNA expression levels in each group were detected via qPCR (Table 4). β-actin was used as the internal reference gene. The detailed primer information is shown in Table 3. All the reactions were performed in triplicate, and the relative quantification of the mRNAs was performed using the 2^−ΔΔCt^ method. The relative quantification of mRNA expression was conducted as described by Livak and Schmittgen (2001) [12].

### 2.8. DRD1 Protein Expression Using a Cellular Immunofluorescence Assay

The expression of the *DRD1* gene protein in the NC, si*DRD1*, and pEGFP-C2 *DRD1* groups was detected after transfection. Identification was performed by immunofluorescence staining with an anti-*DRD1* antibody (1:100).

### 2.9. Measurement of GnRH Release

A sheep GnRH enzyme-linked immunosorbent assay kit (BLUEGENE) was used to assess the GnRH concentration in the cell culture medium 48 h after the transfection of pEGFP-C2-*DRD1* and si*DRD1*-1. The specific procedure was performed according to the manufacturer’s instructions. The concentration of GnRH in each group of samples was calculated using standard curves.

### 2.10. Statistical Analysis

The 2^−ΔΔct^ method was used to analyse the expression level via qRT-PCR, and the experimental data are presented as the mean ± mean. A statistically significant difference was defined as *p* < 0.05. The negative control was used as the control group. RNAi or overexpression of *DRD1* was used to determine the relative changes compared with those in the control group.

## 3. Results

### 3.1. DRD1 Expression in the Kazakh Ewe Hypothalamic–Pituitary–Ovarian Gonadal Axis

**Figure 1:**
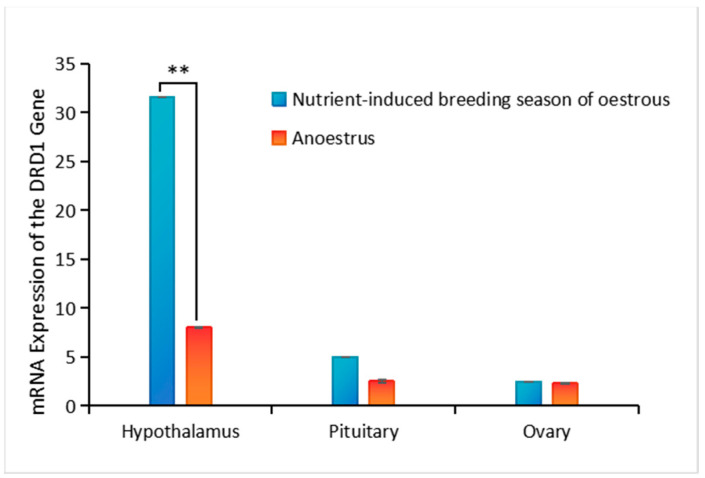
mRNA expression of *DRD1* gene. ** *p* < 0.05.

The hypothalamic–pituitary–ovarian tissue expression profile of the DRD gene in sheep tissues was studied. The *DRD1* mRNA expression in the hypothalamus was significantly greater than that in the pituitary and ovary (*p* < 0.05). Moreover, there was no significant difference in the relative *DRD1* expression between the pituitary and ovary (*p* > 0.05). Taken together, these findings indicate that the *DRD1* gene is highly expressed in the hypothalamus (Figure 1).

### 3.2. Immunohistochemistry of the Hypothalamus, Pituitary, and Ovaries

**Figure 2:**
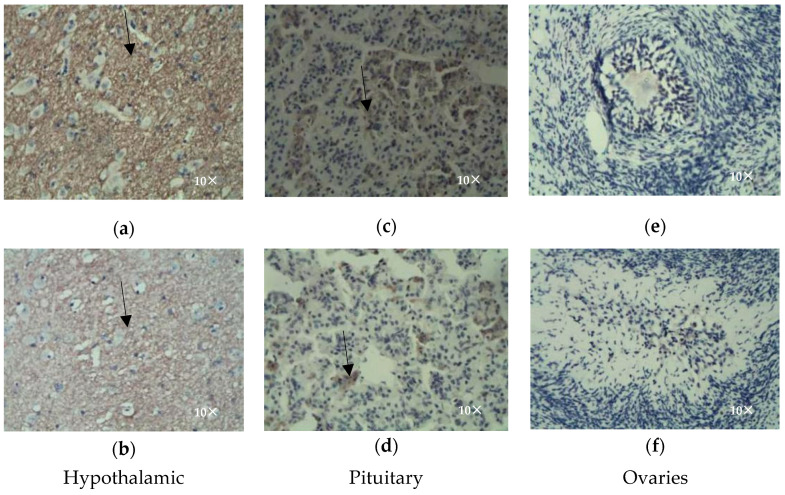
Hypothalamic (**a**,**b**), pituitary (**c**,**d**), and ovarian (**e**,**f**) immunohistochemistry.

Immunohistochemical methods were used to determine the distribution of *DRD1* (Figure 2) in the hypothalamic, pituitary, and ovarian tissues of Kazakh ewes. (1) *DRD1* was highly expressed in the hypothalamic tissue and was expressed on the cell membrane, and a dense network structure of positive cells (brown) was observed after staining. Immunohistochemistry revealed that the oestrous phase had strong positive staining (Figure 2a), while the anoestrous phase had weak positive staining (Figure 2a). (2) A less positive expression was detected in the pituitary gland, with an irregular circular shape and clustered distribution, indicating positive staining of the cell membrane and cytoplasm. Immunohistochemistry revealed that compared with the oestrous phase (Figure 2c) the number of stained positive cells in the anoestrous phase was lower (Figure 2d). (3) After the immunohistochemical staining of the ovarian tissue, *DRD1*-positive cells were not found in the oestrous (Figure 2e) or anoestrous groups (Figure 2f).

### 3.3. Isolation, Culture, and Identification of Primary Hypothalamic Neurons

**Figure 3:**
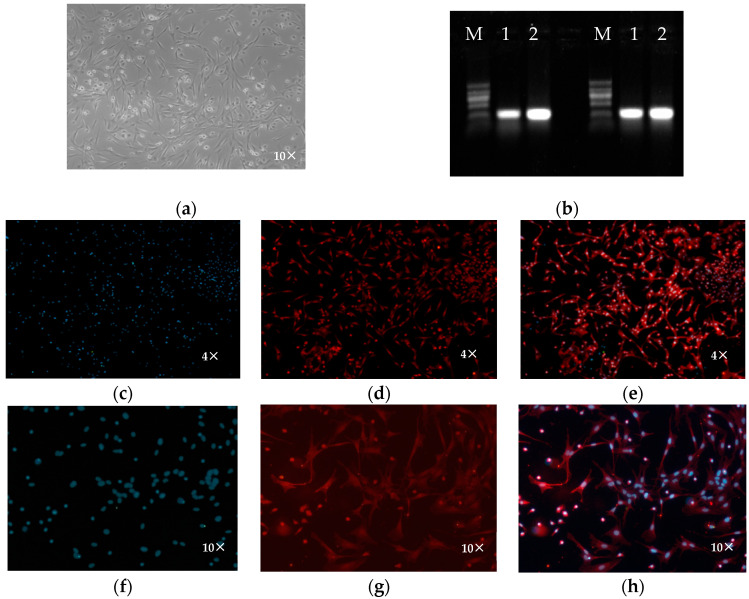
Isolation, culture, and identification of primary hypothalamic neurons. (**a**) Hypothalamic neurons. (**b**) Noggin and TUBA4A gene expression: 1, 2—result of Noggin; M is DNA Marker I. (**c**–**h**) Anti-MAP2-CY3 immunofluorescence.

Hypothalamic neurons were isolated and cultured according to previously described methods [11]. The growth of neurons is slow. After 48 h of isolation and cultivation, the cells were observed, and the medium was changed. The cells met the requirements for subsequent experiments by approximately the 10th day (Figure 3a).

Using the cDNA of hypothalamic neurons cultured for 10 days as a template, we performed PCR amplification of the neuron-specific genes Noggin and TUBA4A to obtain the CDSs of the 168 bp TUBA4A and 144 bp Noggin genes, respectively (Figure 3b).

Immunofluorescence identification was performed on sheep hypothalamic neurons cultured for 10 days using an anti-MAP2-cy3 antibody. Fluorescence microscopy revealed that MAP2 was expressed in the cells (Figure 3c–h), and high-purity hypothalamic neurons were isolated and cultured. After culturing the cells in vitro for 10 days, 100 cells were counted from five fields to determine the purity. The results showed that the percentage of MAP2-positive neurons was 96.1%.

### 3.4. Construction and Validation of Recombinant Plasmids

**Figure 4:**
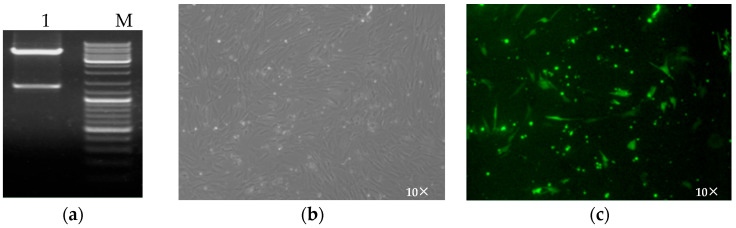
Construction and validation of recombinant plasmids. (**a**) Double enzyme digestion of pEGFP-C2-DRD1. 1: results of double enzyme digestion for pEGFP-C1-DRD1; M: the DNA marker DL10000. (**b**) Inverted microscope image after pEGFP-C2-DRD1 transfection. (**c**) Inverted microscope image after pEGFP-C2-DRD1 transfection.

The recombinant plasmid pEGFP-C2-*DRD1* was subjected to XhoI and BamHI restriction endonuclease digestion, the results of which were consistent with the expected results (Figure 4a). PEGFP-C2-*DRD1* was transfected into sheep hypothalamic neurons using the liposome method [14], and the optimal ratio of plasmids to liposomes was explored to meet the requirements of subsequent experiments. Three groups, namely, the NC, pEGFP-C2, and pEGFP-C2-*DRD1* groups, were established, and the best effect was achieved after 72 h of transfection. The cells were observed under a fluorescence inverted microscope (Figure 4b,c). The complete morphology of the cells and strong fluorescence signals were used for subsequent experiments. After the cells were collected and total RNA was extracted for reverse transcription, qRT-PCR was used to detect the expression levels of the interference genes, and the interference gene β-actin was used as the internal reference. The relative expression level was calculated using the 2-△△CT method. Statistical Product and Service Solutions (SPSS) 17.0 was used for data analysis, and each experiment was repeated three times for each group.

### 3.5. Analysis of DRD1 mRNA Expression in Sheep Hypothalamic Neurons

**Figure 5:**
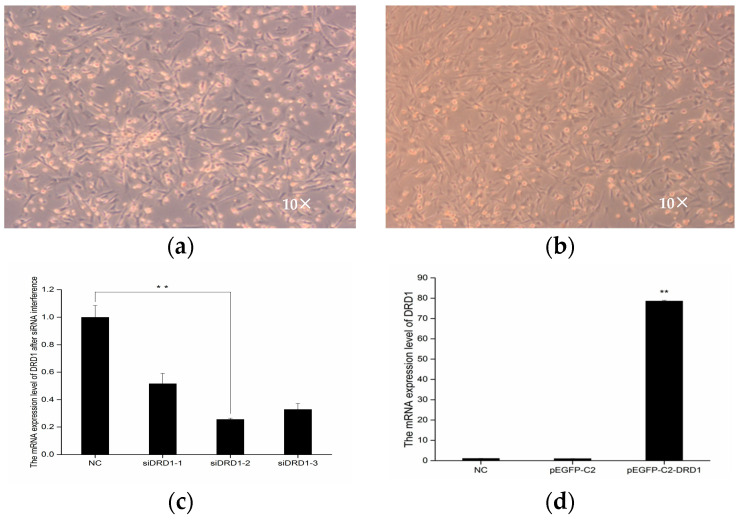
Analysis of DRD1 mRNA expression in sheep hypothalamic neurons. (**a**) Cell morphology before interference; (**b**) blank control group; (**c**) mRNA expression after DRD1 siRNA interference; (**d**) mRNA expression of the DRD1 gene; ** indicates extremely significant differences.

The results of siRNA-*DRD1* transfection into foetal sheep hypothalamic neurons indicated that the interference efficiency of siRNA-*DRD1*-1, siRNA-*DRD1*-2, and siRNA-*DRD1*-3 was 48%, 75%, and 68%, respectively, with siRNA-*DRD1*-2 having the highest interference efficiency (Figure 5).

The mRNA expression level of the *DRD1* gene in the pEGFP-C2-*DRD1* expression vector group was significantly greater than that in the blank control and empty vector groups (*p* < 0.01). The results showed that the *DRD1* gene was highly expressed in hypothalamic neurons.

### 3.6. Analysis of DRD1 Protein Expression

**Figure 6:**
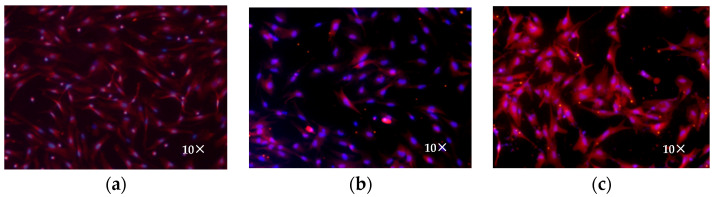
Immunofluorescence staining for detection of DRD1 expression after transfection: (**a**) blank, (**b**) siDRD1, and (**c**) pEGFP-C2-DRD1.

Immunofluorescence was used to detect the expression of *DRD1* in sheep hypothalamic neurons, and *DRD1* was found to be widely expressed in these cells. The expression levels before (Figure 6a) and after interference (Figure 6b) and overexpression (Figure 6c) were measured.

### 3.7. Recombinant Plasmid Transfection into Sheep Hypothalamic Neurons

**Figure 7:**
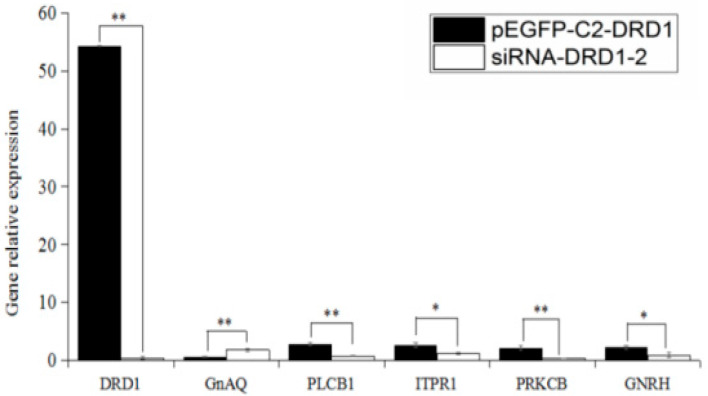
The relative expression of *DRD1* and related genes; ** indicates extremely significant differences, * indicates significant differences.

After the transfection of the sheep hypothalamic neurons, the effects of *DRD1* and related genes were quantitatively detected in real time. Using the untreated group as a control, the relative expression level of *DRD1* in the pEGFP-C2-*DRD1* group after transfection was significantly greater than that in the siRNA-*DRD1*-2 transfection group (*p* < 0.01), while the expression of the downstream gene GnAQ in *DRD1* was significantly lower than that in the interference group (*p* < 0.05), and the expression in the PRKCB overexpression group was significantly greater than that in the interference group. Similarly, the relative expression level of GnRH was significantly greater in the treatment group than in the interference group (*p* < 0.05) (Figure 7).

### 3.8. Effect of DRD1 on GnRH Secretion

**Figure 8:**
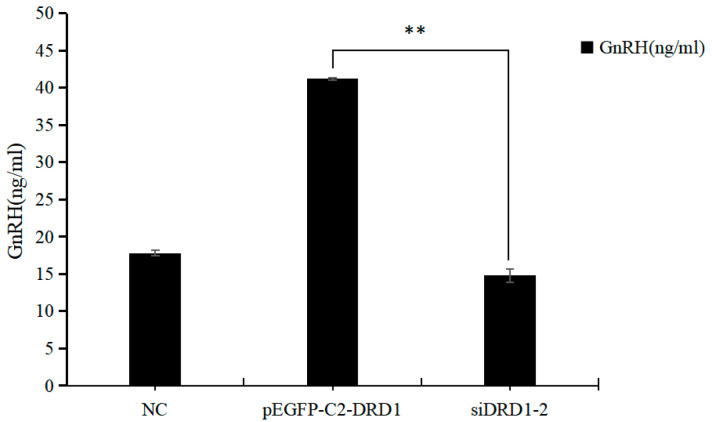
GnRH test results (** indicates extremely significant differences).

An enzyme-linked immunosorbent assay (ELISA) was used to assess the secretion of GnRH in the hypothalamic neurons of the *DRD1*-overexpressing group and si*DRD1*-2 group after transfection. The results showed that the secretion of GnRH in the pEGFP-C2-*DRD1*-transfected group was significantly greater than that in the si*DRD1*-transfected group and blank group (*p* < 0.05), indicating that *DRD1* can affect the secretion of GnRH (Figure 8).

## 4. Discussion

Sheep farming is an important component of China’s animal husbandry industry, and Kazakh sheep are valuable local sheep breeding resources that play important roles in social, economic, and animal husbandry development. Kazakh lambs have excellent quality and unique flavours and are highly favoured by consumers. However, due to their typical seasonal oestrous status, sheep give birth once a year, which seriously restricts the development of the sheep farming industry. The key to vigorously developing efficient breeding in sheep farming is to improve reproductive capacity; therefore, achieving year-round oestrus in sheep has significant production implications.

The seasonal oestrus of sheep is precisely regulated by the hypothalamic–pituitary–gonadal axis (HPGA), which is accompanied by changes in natural lighting and is precisely turned on and off. The difference in the pulse frequency of GnRH is a key factor in the cycle transition between the breeding season and nonbreeding season in ewes. The pulse frequency of GnRH significantly decreases during the nonbreeding season [15], and the fluctuation frequency of GnRH increases during the transition from the nonbreeding season to the breeding season [16,17]. Therefore, if the GnRH concentration does not reach a certain peak, oestrus will not occur. Our previous mRNA-miRNA coanalysis showed that *DRD1* is a differentially expressed gene that may be associated with seasonal oestrus in sheep [9]. Previous studies have shown that sheep hypothalamic neurons can be used as cell models for studying target gene functions and hormone secretion related to seasonal oestrous regulation in sheep [11]. Therefore, we designed our study using sheep hypothalamic neurons to provide insight into the molecular mechanism through which the *DRD1* gene regulates oestrus and its involvement in GnRH secretion regulation.

*DRD1* (dopamine receptor D1) is a G protein family-coupled receptor that binds to dopamine to activate a series of signalling pathways and regulate various physiological functions. Dopamine receptors are widely distributed and have diverse functions in animals. Currently, five types of dopamine receptors, *DRD1*-DRD5, have been successfully isolated, among which the *DRD1* gene is widely expressed in the mammalian brain and plays an important role [18]. At present, research reports on *DRD1* have focused mainly on human pathological phenomena, using mice as models to study neural signal transmission, hormone secretion, growth and development, and neurological disorders involving the *DRD1* gene in the brain [19,20]. It has been confirmed that stimulating *DRD1* promotes the secretion of prolactin to regulate the reproductive performance of birds [21,22]. However, in the present study, the expression of the *DRD1* gene in the hypothalamic, pituitary, and ovarian tissues of Kazakh sheep in oestrus and anoestrus was detected via qRT-PCR and immunohistochemistry. The expression level of the *DRD1* gene was the highest in the hypothalamic tissue, and the expression level in the oestrous group was significantly greater than that in the anoestrous group (*p* < 0.05). The immunohistochemical results showed that during oestrus, *DRD1* was widely expressed in the hypothalamus and strongly positively expressed. In the pituitary gland, it showed weak positive cluster-like scattered expression, with significantly lower expression levels than those in the hypothalamus, and almost no positive staining was observed in the ovaries. During oestrus, *DRD1* is also widely expressed in the hypothalamus but shows weak positivity compared to oestrous staining. Weakly positive cluster-like cells with a scattered expression can be observed in the pituitary gland, and the expression level is significantly lower than that in the oestrous pituitary tissue. Similarly, positively stained cells were almost not observed in the ovaries. The hypothalamus plays an important role in the reproductive regulation of mammals, serving as the “headquarters” of the HPG axis to receive signals and emit them, thereby regulating animal reproductive activities. GnRH is an important regulator of sexual behaviour in mammals. GnRH is synthesised and secreted by the hypothalamus, which is located at the top of the hypothalamic–pituitary–ovarian axis. This study revealed that *DRD1* is spatiotemporally related to the hypothalamic tissue of Kazakh sheep during oestrus and that *DRD1* expression in the hypothalamus is significantly greater than that in pituitary and ovarian tissues. It is speculated that *DRD1* and GnRH secretion in the hypothalamus may be related, which in turn affects sheep oestrus.

We subsequently regulated the expression of the *DRD1* gene in sheep hypothalamic neurons in vitro to study its effect on GnRH secretion. By transfecting the recombinant vectors pEGFP-C2-*DRD1* and siRNA-*DRD1*-2 into sheep hypothalamic cells, it was found that after the upregulation of *DRD1* expression, the expression of the downstream gene *GnAQ* was downregulated, while that of GnRH was significantly upregulated. In the early stage of our research, we showed that miR-200b interference downregulated *GNAQ* gene expression and upregulated GnRH expression in sheep hypothalamic neurons [23], interfered with GnAQ gene expression, and upregulated GnRH expression in sheep hypothalamic neurons [24]. This finding is consistent with the results of the present study. The recombinant vectors pEGFP-C2-*DRD1* and siRNA-*DRD1*-2 were transfected into sheep hypothalamic cells, which indicated that after *DRD1* expression was upregulated, the expression of the downstream gene *GnAQ* was downregulated, while that of GnRH was significantly upregulated. Studies have shown that miR-200b can interfere with the downregulation of *GNAQ* gene expression and the upregulation of GnRH expression in sheep hypothalamic neurons [23,25]. These findings are consistent with the results of the present study. Dopamine is a catecholamine neurotransmitter that helps cells transmit pulses [26,27,28], while tyrosine is a precursor to dopamine synthesis in living organisms [29]. One study reported that the tyrosine treatment of rabbit anterior pituitary cells in vitro promoted LH secretion and enhanced the effect of GnRH [30]. Additionally, L-tyrosine significantly increased the secretion of progesterone in the ovaries of rats and mice and promoted and induced ovulation [31,32,33,34]. In a previous study, sheep hypothalamic neurons were treated with different concentrations of tyrosine, and an appropriate concentration of tyrosine was detected by ELISA to affect GnRH secretion [35]. Our research group revealed that, in the early stage, nutrient induction may share some common pathways with the classical light regulatory pathway. The key shared regulatory point is at the *GnAQ* gene. Moderate supplementation with folic acid upregulates the methylation level of the *GnAQ* promoter region in sheep hypothalamic cells cultured in vitro, inhibits *GnAQ* expression, and promotes GnRH secretion [11]. However, our study focuses only on Kazakh sheep, which may limit the generalisability of the findings to other breeds. Subsequent knock-out and knock-in animal model experiments will be conducted to provide validation for further mechanism theories.

**Figure 9:**
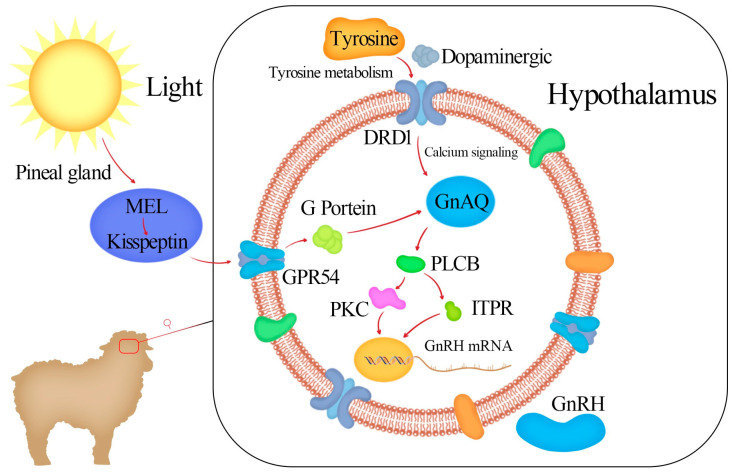
Simple pathway through which DRD1 is involved in GnRH secretion.

Our research revealed that the expression of the *DRD1* gene in the hypothalamic, pituitary, and ovarian tissues of Kazakh sheep during oestrus and oestrus has significant spatiotemporal and tissue specificity. These results indicate that DRD1 mediates the effects on hypothalamic neurons secreting GnRH through dopamine and calcium signalling pathways (Figure 9). This study revealed the role of the DRD1 gene in regulating GnRH secretion in hypothalamic neurons in vitro, laying the foundation for further research on the molecular mechanism through which DRD1 affects seasonal oestrus in sheep.

## Figures and Tables

**Table 1:** Primers for detecting mRNA levels via real-time quantitative PCR.

Primer Name	Primer Sequence (5′–3′)	Tm/°C	Length (bp)
*DRD1* (ENSOARG00020020572)	TCGCCCAGAAACAAATACGG AGAAAGGGAGCCAGCAACAC	54	207
β-actin (ENSOARG00020008714)	AGAGCAAGAGAGGCATCC TCGTTGTAGAAGGTGTGGT	50–68	108

**Table 2:** *DRD1* interference fragments.

siRNA	siRNA Sequence (5′-3′)
siRNA-*DRD1*-1	GCAUUCUCACAGCCUGUUUTT AAACAGGCUGUGAGAAUGCTT
siRNA-*DRD1*-2	CCGCUACAGGUAACGGAAATT UUUCCGUUACCUGUAGCGGTT
siRNA-*DRD1*-3	GGGCUAAUUCCUCCUUGAATT UUCAAGGAGGAAUUAGCCCTT

**Table 3:** The primer sequences.

Primer Name	Primer Sequence (5′–3′)	Size/bp	Tm/°C
TUBA4A (NC_019468.2)	GAGGATGCCGCTAACAAC CAGTGAGGTGAAGCCAGAG	168	55
Noggin (NM_001163055.2)	TGCCGAGCGAGATCAAAGC CAGGTCGTTCCACGCATACAG	146	59

**Table 4:** Primers for detecting caspase-9 mRNA levels via real-time quantitative PCR.

Primer Name	Primer Sequence (5′–3′)	Tm/°C	Length (bp)
*DRD1* (ENSOARG00020020572)	TCGCCCAGAAACAAATACGG AGAAAGGGAGCCAGCAACAC	54	207
*GnAQ* (ENSOARG00020004509)	GAGAACCGAATGGAGGAAAG GAAATAGTCAACTAGGTGGGAAT	54	144
*ITPR1* (ENSOARG00020012519)	GTCCGCCACCAGTTCAA CCTCTGCTGCTAAATAATGC	54	134
*PLCB1* (ENSOARG00020023836)	TTTTCCGAATTTGGTGC GGCGAGGCTGTTGTTAG	54	171
*PRKCB* (ENSOARG00020025105)	GATCGAACTACACGGAACG TAGAGGCTCAGTGGTAAAGAAT	54	203
*β-actin* [13] (ENSOARG00020008714)	AGAGCAAGAGAGGCATCC TCGTTGTAGAAGGTGTGGT	50-68	108

## Data Availability

All data contains in the paper.
